# Effects of short-term pretreatment with atorvastatin on mobilization of hematopoietic progenitor cells: A double-blind, randomized, controlled trial

**Published:** 2015-10-01

**Authors:** Mehdi Mohammadi, Mohammad Vaezi, Bahram Chahardouli, Molouk Hadjibabaie, Ardeshir Ghavamzadeh

**Affiliations:** 1 Clinical Pharmacy Department, Faculty of Pharmacy, Tehran University of Medical Sciences, Tehran, Iran; 2 Hematology-Oncology and Stem Cell Research Center, Tehran University of Medical Sciences, Tehran, Iran; 3 Research Center for Rational Use of Drugs, Tehran University of Medical Sciences, Tehran, Iran

**Keywords:** Atorvastatin, Autologous, HSCT, Mobilization

## Abstract

**Background:** Despite recent advances in mobilization techniques, a considerable portion of patients fail to mobilize sufficient number of cells for successful autologous stem cell transplantation. There are several studies available that have demonstrated enhanced mobilization of endothelial progenitor cells with atorvastatin. Therefore, this prospective trial was conducted to evaluate the mobilizing effect of atorvastatin on hematopoietic progenitor cells.

**Subjects and Methods:** Forty-four autologous HSCT candidates were randomized in a double-blind controlled trial to receive atorvastatin 40 mg daily or placebo plus standard G-CSF regimen. Treatment was initiated at the time of hospitalization and continued until the day of cell harvest. Independent-samples T-Test, Repeated Measures ANOVA and Mann-Whitney U test were performed to compare means. Categorical variables were analyzed using Chi-square and Fisher’s exact test.

**Results:** Mean number of hematopoietic progenitor cells per microL of peripheral blood at the time of cell harvest did not differ significantly between the two groups. There was no statistically significant difference in secondary outcomes like time of platelet or PMN engraftment, occurrence of bleeding or infectious episode, duration of hospitalization and etc.

**Conclusion:** The results of this study did not support beneficial effects of atorvastatin on mobilization of hematopoietic progenitor cells from bone marrow.

## Introduction

 Hematopoietic stem cell transplantation (HSCT) has been used to treat a variety of malignant and non-malignant disorders.^        1 ^ The success of the procedure has increased dramatically by using new approaches to supportive care.^   2 ^ Peripheral blood has supplanted bone marrow as the main source of stem cells in both autologous and allogeneic HSCT. This transition skips the need for general anesthesia and multiple aspirations of bone marrow.^        3 ^ There are some reports that different outcomes may be anticipated by altering the source of stem cells. Compared with bone marrow, transplantations performed using peripheral blood-derived stem cells have been associated with reduced incidence of disease relapse at the expense of more severe acute graft-versus-host disease. 4^,^^5^  However, peripheral blood yields a greater number of progenitor cells which results in hastened immune recovery and subsequent protection against infections.^        6 ^ Mobilization of progenitor cells from bone marrow by G-CSF ± chemotherapy has been the standard of care in both patients and normal donors.^   7 ^ Despite advances that have been made in mobilization techniques, failure rate is still considerable. Lower number of infused cells results in delayed engraftment and associated higher cost to health care system.^        8 ^

A variety of disease- or patient-related factors has been associated with mobilization failure including but not limited to monotherapy with G-CSF.^        9 ^ Therefore, attempts have been made to potentiate the efficacy of G-CSF by adding newer mobilizing agents.^        8 ^ Plerixafor is one of the most promising mobilizing agents marketed recently and has been effective either as primary or secondary regimen when combined with G-CSF.^10^^,^^11^ 

Unfortunately, the high cost of medication 12 and its limited availability in many countries have led to restricted use of plerixafor in many clinical settings. Therefore, ongoing search is required in this area to find new agents that are effective and easy to access. 3-hydroxy-3-methylglutaryl coenzyme A reductase inhibitors or statins have been shown repeatedly to enhance the mobilization of endothelial progenitor cells (EPCs). ^13^^-^^15^  A retrospective study has also discussed the potential for such beneficial effects on hematopoietic progenitor cells (HPCs).^16^

Considering favorable effects observed in these trials, it was hypothesized that statin co-administration with standard treatment might enhance hematopoietic progenitor cell mobilization and apheresis yield. To the best of our knowledge, no prospective study has examined this effect. Therefore, the goal of present study is to evaluate the effect of atorvastatin on mobilization of HPCs.

## SUBJECTS AND METHODS

**Study design**

 This randomized, placebo-controlled, double-blind trial was conducted between November 2013 and August 2014 at the Hematology-Oncology and Stem Cell Research Center (HOSCRC) of Shariati Hospital affiliated with Tehran University of Medical Sciences. The study design was reviewed and confirmed by the Ethics Committee of HOSCRC. (No: 8911358006-135492)

**Inclusion and exclusion criteria**

All autologous HSCT candidates with primary diagnoses of Hodgkin’s lymphoma (HL), non-Hodgkin’s lymphoma (NHL) or multiple myeloma (MM) that aged at least 18 years old and endorsed the informed consent were recruited. Patients who were taking a statin drug (lovastatin, simvastatin, atorvastatin, fluvastatin and rosuvastatin) at the time of enrollment or developed suspected drug- related toxicity during treatment were excluded from the study.

**Allocation and procedure**

Forty-four patients were allocated to either intervention or placebo group using permuted block randomization model. Randomization was done by an independent person. Patients in the intervention group received atorvastatin (Abidi Pharmaceutical Co., Tehran, Iran) 40 mg daily initiated at the time of hospitalization and continued until the day of apheresis. Patients in the control group received placebo (Abidi Pharmaceutical Co., Tehran, Iran) under the same schedule. All patients received G-CSF according to the ward protocol.

For patients diagnosed with HL or NHL, this regimen consisted of G-CSF 300 mcg daily for days -9 to -6 (days 9 to 6 before infusion of hematopoietic stem cells), 600 mcg at day -5 and 900 mcg at day -4. For those patients affected with MM, the regimen consisted of G-CSF 300 mcg at days -6 to -4 and 600 mcg at day -3. G-CSF was administered as subcutaneous injections throughout study. All patients underwent apheresis at the same time of the day (early morning). Physicians, patients, nursing staff and data analyzer were all blind to the allocations. Liver function tests and CPK level were measured for all patients at baseline.

 Repeated LFT or CPK measurements were performed in cases of suspected drug-related toxicity and treatment was held if clinically indicated. Liver injury was defined as symptomatic ALT elevations more than 3-fold of upper limit of normal levels (ULN) or asymptomatic elevations more than 5 times the ULN.^   17 ^ Muscle injury was defined as presence of muscle symptoms with or without elevated CPK levels.^        18 ^ Platelet engraftment was defined as the first of three consecutive days without platelet transfusion with platelet counts > 20,000/mm^3^ after transplantation, while PMN engraftment was defined as the first of three consecutive days with PMN count > 500/mm^3^ post-transplantation. Duration of hospitalization started from the day of stem cell infusion until discharge.

**Sample preparation and analysis**

All patients had peripheral blood samples drawn for flow cytometry at baseline (prior to first dose of atorvastatin or placebo) and just before apheresis. Obtained samples were immediately analyzed by flow cytometry (Partec PAS-III, Partec, Germany). A triplet panel of surface markers including CD34 (PE-labeled, Exbio, Czech Republic), CD45 (PerCP-labeled, Exbio, Czech Republic) and CD31 (FITC-labeled, Exbio, Czech Republic) was used to characterize HPCs. Cells presenting CD34^+^/CD45^+^/CD31^-^ phenotype were considered as HPC. ^19^^-^^21^ 

**Statistical analysis**

In our center, the average number of CD34^+^ cells harvested during the preceding year was 2.3 × 10^6^ ± 2.0 × 10^6^ cells/kg of patients’ body weight. We assumed equal variances for study patients and HSCT population in our center. We considered a type 1 error of 0.05, statistical power of 80%, and an expected increase of at least 75% in HPC count with atorvastatin. A sample size of 22 patients in each arm was calculated.

Data analysis was performed using SPSS statistics software (Version 19.0. IBM Corp. Armonk, NY). Shapiro-Wilk and Kolmogorov-Smirnov tests were performed to assess normal distribution of continuous variables. Independent-samples T-Test, Repeated measures ANOVA and Mann-Whitney U test were performed to compare means. Categorical variables were analyzed using Chi-square and Fisher’s exact test. Kaplan-Meier estimation and log-rank test was used to compare PMN and platelet engraftment rate between the study groups. A P-value less than 0.05 was considered a significant difference in all tests.

## Results

**Figure 1:**
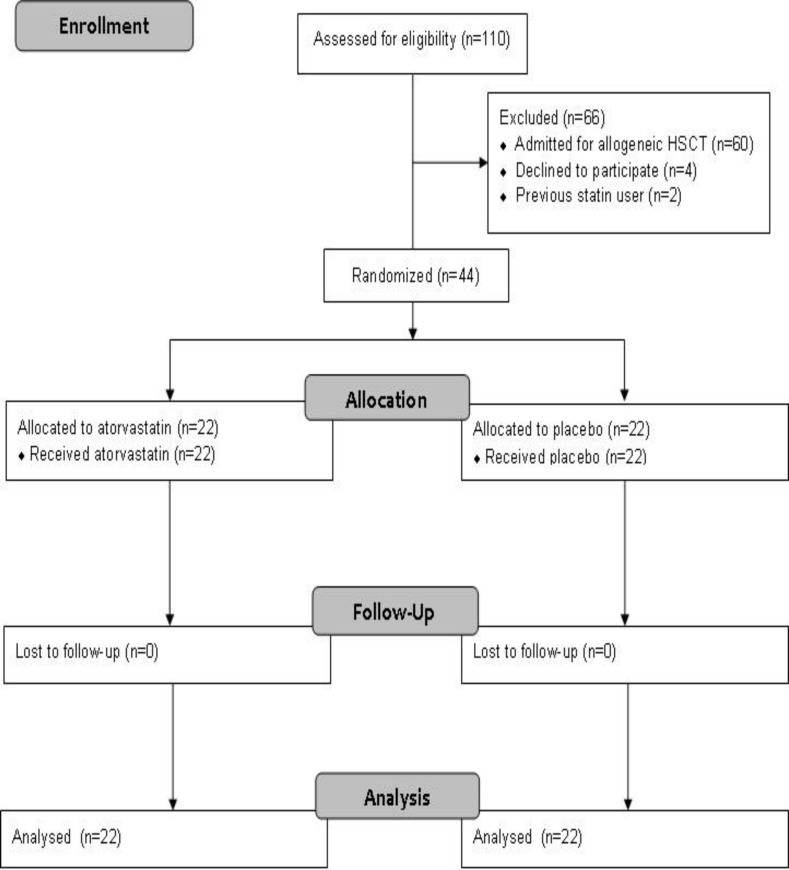
CONSORT flow diagram of study participants

 One hundred and ten patients were assessed for eligibility criteria. Those admitted for allogeneic HSCT (n=60), those who did not sign informed consent form (n=4) or were on previous treatment with any statin agent (n=2) were excluded from participation. Forty-four patients entered the study and no patient was excluded from final analysis (Figure 1).

Demographic and baseline characteristics of participants are described in Table 1. There was no significant difference between two groups in terms of gender, age, weight, primary diagnosis and complete remissions (CR). Total dose of G-CSF per kg of body weight prior to apheresis did not differ significantly between groups (28.5 ± 7.0 vs. 28.9 ± 10 mcg/kg for atorvastatin and placebo group, respectively, p=0.87). Duration of treatment with atorvastatin/placebo was also similar for both groups (5.4 ± 2.9 vs. 6.0 ± 4.2 days respectively, p=0.57). 

**Figure 2:**
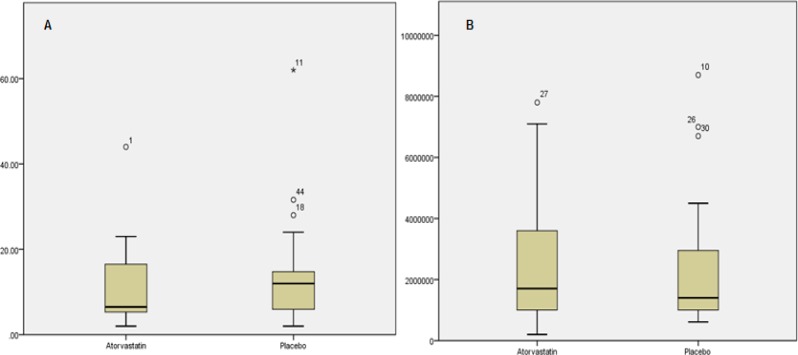
Comparison of total CD34+ cell counts in peripheral blood between two groups. There was no significant difference between two groups in terms of total number of CD34+ cells per microliter of peripheral blood either before (A) or after (B) administration of mobilization regimen

Mean number of HPCs at baseline and harvest time is summarized in Table 2. In general, there was a trend toward placebo group, but the difference did not reach statistical significance. Figure 2 depicts comparisons between two groups regarding total number of CD34^+^ cells and HPCs. Outcomes related to platelet and PMN engraftment are presented in Table 3. In general, there was no difference between two groups regarding engraftment rates or times needed to achieve platelet or PMN engraftment.

**Table 1:** Baseline characteristics of study participants

		**Atorvastatin** **(n=22)**		**Placebo** **(n=22)**	**p-value**
**Age (mean ± SD)**			42.7 ± 12.6	51.1 ± 11.2	0.33
**Male gender, no. (%)**			11 (50)	16 (72.7)	0.13
**Diagnosis No. (%)**	MM		11 (50)	16 (72.7)	0.14*
	HL		9 (40.9)	2 (9.1)	
	NHL		2 (9.1)	4 (18.2)	
**CR No. (%)**	CR1		11 (50)	17 (77.3)	0.07**
	CR2		6 (27.3)	4 (18.2)	
	CR3		3 (13.6)	1 (4.5)	
	CR4		1 (4.5)	0 (0)	
	CR5		1 (4.5)	0 (0)	
**Weight, kg (mean ± SD)**			78 ± 13.4	73.3 ± 15.5	0.28

* p-value represents MM vs. Lymphom

** p-value represents CR1 vs. CR2 or higher

**Table 2:** Comparison of mean number of total CD34+ cells and HPCs between groups

	**Atorvastatin**	**Placebo**	**p-value**
**Total number of harvested CD34 + cells/kg**	2.54 × 10^6 ^± 2.2 × 10^6^	2.44 × 10^6 ^± 2.2 × 10^6^	0.89
**Baseline HPCs** **(/microL of peripheral blood)**	3.4 ± 3.1	3.6 ± 2.4	0.82
**Harvest-time HPCs** **(/microL of peripheral blood)**	10.9 ± 9.7	14.3 ± 13.0	0.33

**Table 3:** Comparison of participants in terms of engraftment

	**Atorvastatin**	**Placebo**	**P- value**
**Occurrence of platelet engraftment at hospital, no (%)**	10/22 (45.5%)	12/22 (54.5%)	0.46
**Time to platelet engraftment, Mean ± SD (days)**	13.9 ± 6.6	14.6 ± 2.6	0.66
**14-day estimated rate of platelet engraftment**	0.6 ± 0.12	0.58 ± 0.12	0.72
**Occurrence of PMN engraftment at hospital**	19/22 (86.4%)	19/22 (86.4%)	1.00
**Time to PMN engraftment, Mean ± SD (days)**	14.2 ± 4.3	13.9 ± 2.3	0.79
**14-day estimated rate of PMN engraftment**	0.65 ± 0.1	0.5 ± 0.1	0.94

Secondary outcomes of study consisted of need for transfusion to maintain homeostasis, occurrence of an infectious or bleeding episode, total dose of G-CSF needed to promote engraftment, duration of hospitalization and mortality. No difference in secondary outcomes was observed between two groups (Table 4).

The drug was well tolerated in all patients. Only one patient who developed liver injury after taking the last dose of drug on the day of apheresis was allocated to intervention group. Since treatment course had already been completed for this patient, he was not excluded from final analysis.

## Discussion

 Clinicians practicing HSCT often encounter patients with poor apheresis yields. Tumor infiltration of bone marrow, advanced age, prior intensive chemotherapy, and prior treatment with some chemotherapeutic agents like melphalan, fludarabine and lenalidomide are risk factors frequently stated to be associated with poor mobilization capacity. 

Even in healthy donors, up to 5% fail to provide sufficient cells. At least 2 × 10^6^ CD34^+^cells per kg of body weight are needed for a successful engraftment in an autologous setting with more prompt engraftments if more than 5 × 10^6^ cells per kg can be harvested safely.^   22 ^ Attempt to find newer agents for mobilization is the subject of an array of ongoing trials. As far as we know, this is the first prospective trial evaluating the mobilizing effect of atorvastatin on HPCs. The results of this study did not show beneficial effects of atorvastatin on mobilization of HPCs, but there are some points that deserve attention. First, the precise “kinetics” of stem cell mobilization with atorvastatin has not been determined yet. Trials performed on EPCs have reported various time frames from initiation of drug until appearance of desired cells in peripheral circulation. At one end of this spectrum, Hibbert et al. reported that even 3 days of treatment with atorvastatin increases peripheral blood count of EPCs, ^        23 ^ while at the other end, this mobilization only has been observed in a 4-month treatment course.^        24^

More diverse intervals have been reported by other studies. ^25^^-^^28^  There is even less information available about HPCs since the only paper proposing such an effect analyzed patients on long-term statin treatment. Duration of treatment with atorvastatin in this trial was approximately 6 days. It may be possible that initiating the drug from an earlier time prior to apheresis can reveal some positive effects. The second point to consider is the possible dose-effect relationship. Pleiotropic effects reported with these agents are commonly associated with higher doses.^   29 ^ Trials conducted in acute coronary syndrome setting have also used moderate to high doses in ranges of 40-80 mg daily. It is not clear now whether such a relationship applies to HPC mobilization. Finally, accurate characterization of cell population of interest may not be possible using immunophenotyping methods.

In this study, CD31, a surface marker of EPCs, was used to rule out contribution of mobilized endothelial progenitors. Given that various panels of surface markers have been proposed for EPCs,^        30 ^ accurate identification of HPCs cannot be ensured. While the total number of CD34^+^ cells was non-significantly higher in intervention arm, mean number of HPCs favored placebo group. This could simply point to preferential mobilization of EPCs rather than hematopoietic counterparts.

No significant difference was observed in secondary outcomes between the study groups. Occurrence and rapidity of platelet and PMN engraftment are functions of the number of infused HPCs; therefore, alteration in these outcomes could not be expected. Rate of bleeding or infectious episodes was not different between two groups. Apart from the inability of atorvastatin to mobilize more HPCs into peripheral circulation, it seems that the function of these cells has not been affected and the study groups were similar in their requirements for packed RBC or platelet transfusion.

The main limitation of this study was relatively small sample size. Therefore, the results of this study cannot be generalized to all patients. Furthermore, subgroup analysis with special look at higher CRs or certain primary diagnoses was not possible because of very small number of patients in related subgroups.

## CONCLUSION

 The results of this study could not demonstrate any beneficial effect of short-term atorvastatin on mobilization of HPCs.
